# Optimal duration of whole-body cryostimulation exposure to achieve target skin temperature: influence of body mass index—a randomized cross-over controlled trial

**DOI:** 10.1186/s40101-024-00375-2

**Published:** 2024-11-01

**Authors:** Hela Jdidi, Claire de Bisschop, Benoit Dugué, Romain Bouzigon, Wafa Douzi

**Affiliations:** 1 Laboratory “Mobilité, Vieillissement, Exercice (MOVE)—UR 20296”, Faculty of Sport Sciences, University of Poitiers, Poitiers, 86000 France; 2 Department of Sport and Performance, Unit of Formation and Research in Sports, Laboratory C3S (EA 4660), University of Franche-Comte, Besançon, 25000 France; 3 Inside the Athletes 3.0, Sports Performance Optimization Complex, Besançon, 25000 France

**Keywords:** Whole-body cryostimulation, Cold exposure, Skin temperature, Body mass index, Body fat

## Abstract

**Background:**

The efficacy of whole-body cryostimulation (WBC) may be influenced by individual characteristics. The aim of this study is to determine the optimal exposure time required to reach the analgesic threshold of 13.6 °C, which has been proposed to be a target temperature to be reached at skin level. Our objective is also to follow the skin temperature changes during and after WBC considering the participants body mass index (BMI).

**Methods:**

Thirty healthy men were assigned into 2 groups based on their BMI [normal weight (*n* = 15; BMI = 21.53 ± 1.63 kg·m^−2^) and overweight (*n* = 15; BMI = 27.98 ± 1.16 kg·m^−2^)]. In a random order, each participant experienced a 4-min WBC exposure, as well as a control session with no cold exposure. Skin temperature was measured using a thermal imaging camera during and after cold exposure.

**Results:**

Normal weight participants reached the threshold in 4 min, whereas overweight participants reached it in 3 min 30 s. Following WBC, a rapid mean skin temperature (MsT°) increase was observed for both groups, immediately after exposure. However, after 30 min, MsT° remained significantly lower than at baseline.

**Conclusion:**

Our findings suggest that appropriate WBC dosage may differ according to BMI. Understanding the impact of such variable on cold exposure outcomes can help to optimize WBC treatments and maximize potential benefits.

## Introduction

Whole-body cryostimulation (WBC) is defined as a body cooling technique that consists of brief exposure of the entire body to extremely cold temperatures [1]. It is mainly used to reduce pain, fatigue, inflammation, oxidative stress, and muscle soreness after physical exercise and to improve sleep quality [2, 3]. The use of WBC is considered as an intense stressor that triggers immediate regulatory responses in the human body and elicits short-term physiological mechanisms [1]. Temperature variations are detected at the cutaneous level through thermoreceptors. These changes stimulate a range of physiological regulatory mechanisms aimed to maintain a constant core body temperature [4].

The effectiveness of WBC in eliciting physiological responses can be influenced by several variables, including the duration, frequency, and temperature of the exposure [5]. However, in this context, there is a lack of clear guidance on the optimal duration of a single session. While existing scientific literature suggests that a typical WBC session lasts between 2 and 5 min [6], the rationale behind choosing longer or shorter exposure times remains unclear. For instance, Fonda et al. expressed that there is no need to perform sessions longer than 2 min 30 s [7]. Selfe et al. suggested that a minimum exposure duration of 2 min was required to induce physiological changes in core and skin temperature, as well as thermal sensation responses [8]. Considering the importance of skin temperature as physical attribute, it serves as a diagnostic parameter in various medical and sporting settings [9]. Indeed, the magnitude of skin tissue cooling attained through cryotherapy may play a critical role in inducing analgesic effects [10]. In this study, we chose the suggested threshold for skin temperature based on extensive prior research and clinical observations. This threshold was chosen to balance therapeutic efficacy with participant safety (e.g., frozen tissues), maximizing the benefits of cryostimulation without compromising health [11]. Scientific evidence suggests that reaching a critical level of tissue cooling is essential to optimize the effectiveness of cold therapy. Specifically, maintaining skin temperatures below 13.6 °C is required to trigger significant cold-induced analgesia [12]. This analgesic effect seems to occur through various mechanisms, including reduced receptor sensitivity and decreased nerve conduction velocity (NCV) [13]. Previous studies indicate that skin temperature of approximately 13.6 °C is required to achieve a 10% reduction in NCV, which is considered optimal for clinical analgesia [14]. By adhering to the 13.6 °C threshold, we aimed to ensure participant safety while accurately determining the optimal duration of exposure. The extent of skin cooling achieved during cryotherapy exposure directly affects its ability to induce beneficial cold responses and avoid effects. This information underscores the importance of precisely managing skin temperature levels to optimize the efficacy of cold exposure while minimizing potential drawbacks. The difficulty lies in the fact that prolonged cooling can lead to health risks, while insufficient duration may fail to induce beneficial responses [10]. Therefore, it is of importance to assess the duration of WBC exposure.

Indeed, the efficacy of WBC may be influenced by several factors, including body mass index (BMI) as lean and overweight individuals exhibit a distinct response to cold stimuli [15–17]. Observed variations may be linked to the insulating properties of body fat [18] and to the skin vasoconstrictive reaction to cold exposure [19]. It has been suggested that body fat percentage is positively correlated with skin temperature decrease, highlighting the role of body fat percentage as a contributing factor to skin temperature magnitude [9, 20]. These features emphasize the importance of considering BMI, body composition, and fat distribution when evaluating the efficacy and potential benefits of WBC.

Our hypothesis suggests that to achieve equivalent values of skin temperatures, the optimal exposure duration may differ between participants of different BMI categories. Therefore, our objective is to evaluate changes in skin temperature considering BMI and to determine the optimal exposure time required to attain the analgesic threshold of 13.6 °C at the skin level. Therefore, our study may provide valuable insights into optimizing the application and effectiveness of WBC.

## Materials and methods

### Participants

Thirty physically active and healthy men volunteered to participate in this study. The ethnicity of the participants was Caucasian. None was accustomed to cold exposure or any cryostimulation treatments. The participants were divided into two groups based on their BMI: normal weight (*n* = 15) and overweight (*n* = 15) (see Table 1 for characteristics). According to the World Health Organization (WHO), individuals with a BMI between 18.5 and 24.9 kg·m^−2^ are classified as having a normal weight (normal weight group), while those with a BMI between 25 and 29.9 kg·m^−2^ are considered overweight (overweight group) [21]. Given that body mass may influence the thermal response to cryostimulation, body fat percentage was controlled in addition to BMI categorization. According to the American Council on Exercise, for normal weight participants, the body fat percentage was less than 17%, and for overweight participants it ranged from 18 to 25% [22]. This approach allowed us to avoid including individuals who might be falsely categorized as overweight due to a high BMI and a low percentage of body fat. Participants were checked for contraindications to cold exposure, such as cold hypersensitivity (Raynaud’s syndrome), cold allergy, acute infection, and history of heart disease or circulatory pathologies. None of them was on medication. Participants were asked to abstain from alcohol, coffee, and strenuous physical exercise 2 h before and 30 min after the intervention. They received an oral and written explanation of the study protocol, the risks and benefits associated with the study’s experimental procedures according to the Declaration of Helsinki. Additionally, they signed a written informed consent. The study was approved by the National Ethics Committee (South-East I Committee for the Protection of Persons; number: 2022-A00525-38).

**Table 1:** Participant characteristics

	Normal weight group	Overweight group
*N*	15	15
Age (years)	21.07 ± 2.12	22.20 ± 3.08
Height (m)	1.79 ± 0.07	1.77 ± 0.05
Weight (kg)	69.01 ± 9.24	87.93 ± 5.32†
BMI (kg·m^−2^)	21.53 ± 1.63	27.98 ± 1.16†
Body fat percentage (%)	14.63 ± 1.82	20.77 ± 2.40†

Data are presented as mean ± SD

†Significant difference between the groups (*p* < 0.001)

### Experimental design

**Fig. 1:**
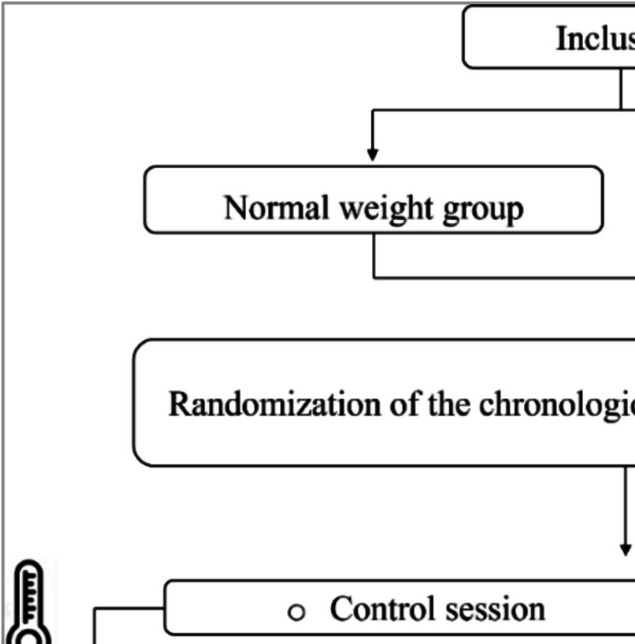
Study design. WBC whole-body cryostimulation

In a random order, each participant experienced 4 min of WBC exposure as well as a control session with no cold exposure (Fig. 1). Sessions were conducted at the same time of the day and separated by a 7-day interval. Cold exposure sessions were administered in a WBC chamber consisted of two compartments. The participant initially spent 30 s in the first adaptation room at − 25 °C, before entering the main compartment at an exposure temperature of − 50 °C with a mean wind speed of 2.3 m/s (WBC chamber, Aurore Concept, Noisiel, France) [23]. The WBC chamber used in this study generates dry, cold air using advanced forced convection technology. Specifically, it employs electrically powered frontal unilateral wind. This method of generating cold air does not involve the use of liquid nitrogen. Participants alternated each 30 s facing a fan and then exposing their back, continuously until the end of the session, to ensure a uniform cooling of the anterior and posterior sides. During exposure, a minimal amount of clothing (shorts, socks, shoes, gloves, and a head cover) was worn to avoid frost bite injury. A surgical mask was also worn.

Skin temperature was measured before, immediately after, and every 5 min during a 30-min period after the intervention. During the entire exposure period, skin temperature measurements were taken at 30-s intervals. Control sessions were conducted under stable ambient temperature conditions where identical measurements were taken. During the control session, participants wore the same clothing during both the control and WBC trials to ensure consistency. After the WBC sessions or the control session, participants sat in a room at ambient temperature, wearing only shorts. The ambient room temperature where the experiments were conducted was 20.4 ± 1.5 °C. Participants were instructed to refrain from excessive movement to avoid warming up their bodies. The experiments were conducted over 3-month period, from December to February, which corresponds to the winter season in our country.

### Measurements

#### Anthropometric measures

Precise height measurements were obtained using a wall stadiometer 1 week prior to the experimental protocol. Additionally, bodyweight and composition were assessed using a validated 8-point bio-impedance device (Tanita BC418-MA, Tanita Corp., Tokyo, Japan).

#### Skin temperature

Skin temperature was measured with a thermal imaging camera (Optrix GmbH, Holzkirchen, Germany). The camera was positioned at a distance of 1.5 m away from the participant. The emissivity was set at 0.98. A lens with a focal length of 80 mm was installed on the camera. Data collection was implemented using the Optrix PI Connect software. Additionally, skin temperature within the cryotherapy chamber was monitored using a pre-installed thermal camera of the same technology. To calculate the mean skin temperature (MsT°), we recorded the average skin temperatures of four distinct body segments: the trunk, arm, thigh, and leg. Both anterior and posterior areas were considered. The body was segmented into polygon-shaped zones, allowing for precise delineation of the regions of interest. The Optrix PI Connect software provided the mean skin temperature for each zone within the designated polygon. These segment-specific mean temperatures were then used to calculate the overall mean skin temperature using a weighted formula. The MsT° of the entire skin body is calculated using the Ramanathan formula [24]: MsT° = 0.3 MsT° trunk + 0.3 MsT° arm + 0.2 MsT° thigh + 0.2 MsT° leg.

### Statistical analysis

The results are reported as mean values ± standard deviation. The Shapiro–Wilk test was used to test whether the distribution was Gaussian. A 2-way repeated measures analysis of variance (ANOVA) was conducted to examine skin temperature variation during cold exposure (time × group) and MsT° analysis based on body region (body area × group). A 3-way ANOVA (time × group × condition) was used to assess skin temperature variation following WBC exposure. When a significant interaction between factors was found, post hoc analyses were performed using the Bonferroni test. The level of significance for all tests was set at *p* < 0.05. Pearson test was used for correlation analysis between variables. STATISTICA 10.0 software (StatSoft, Inc., Tulsa, OK, USA) was used for the analysis.

## Results

### Mean skin temperature changes during WBC exposure

**Fig. 2:**
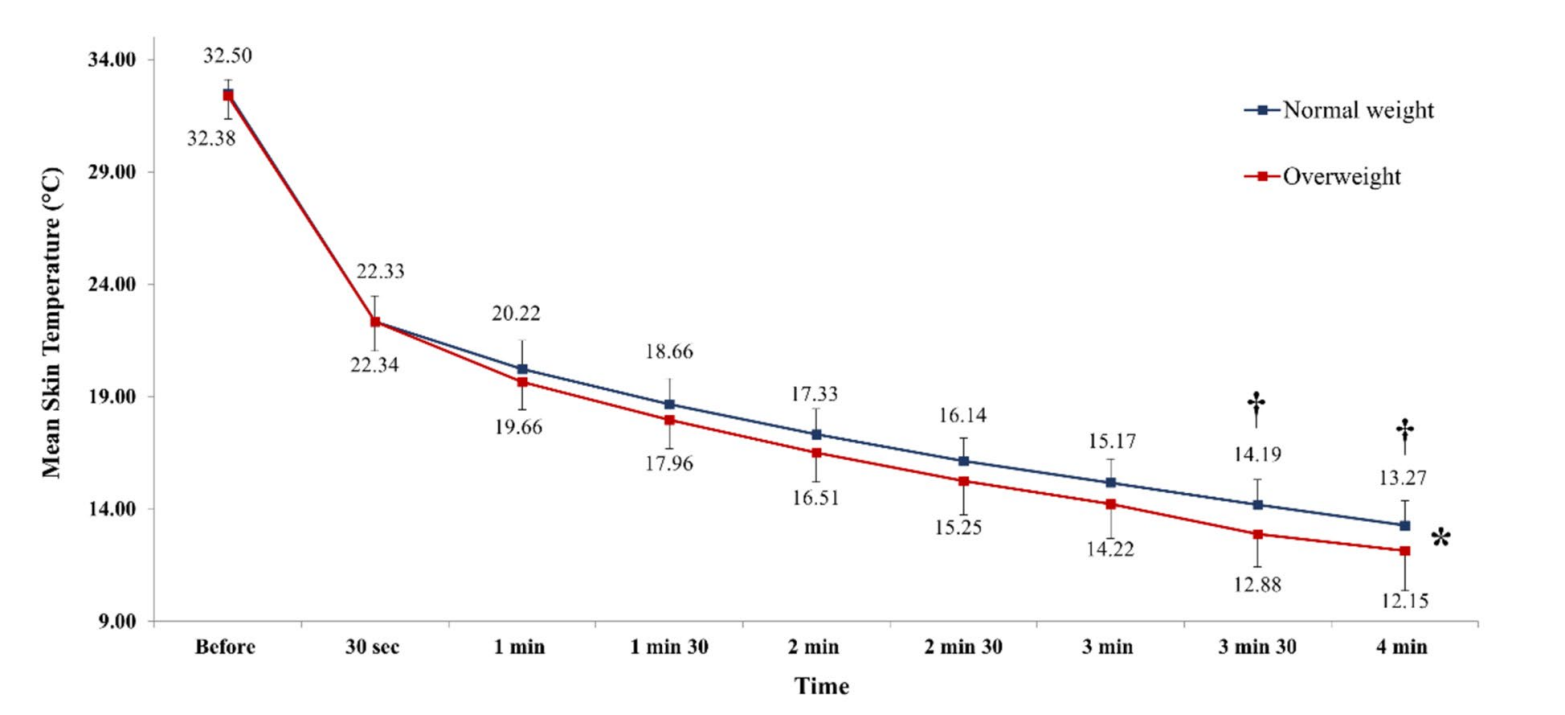
BMI effect on mean skin temperature during WBC exposure. *: Significant difference compared with baseline at all measurement times, in both groups (*p* < 0.001); †: significant difference between groups (*p* < 0.05); data are presented as mean ± SD

**Fig. 3:**
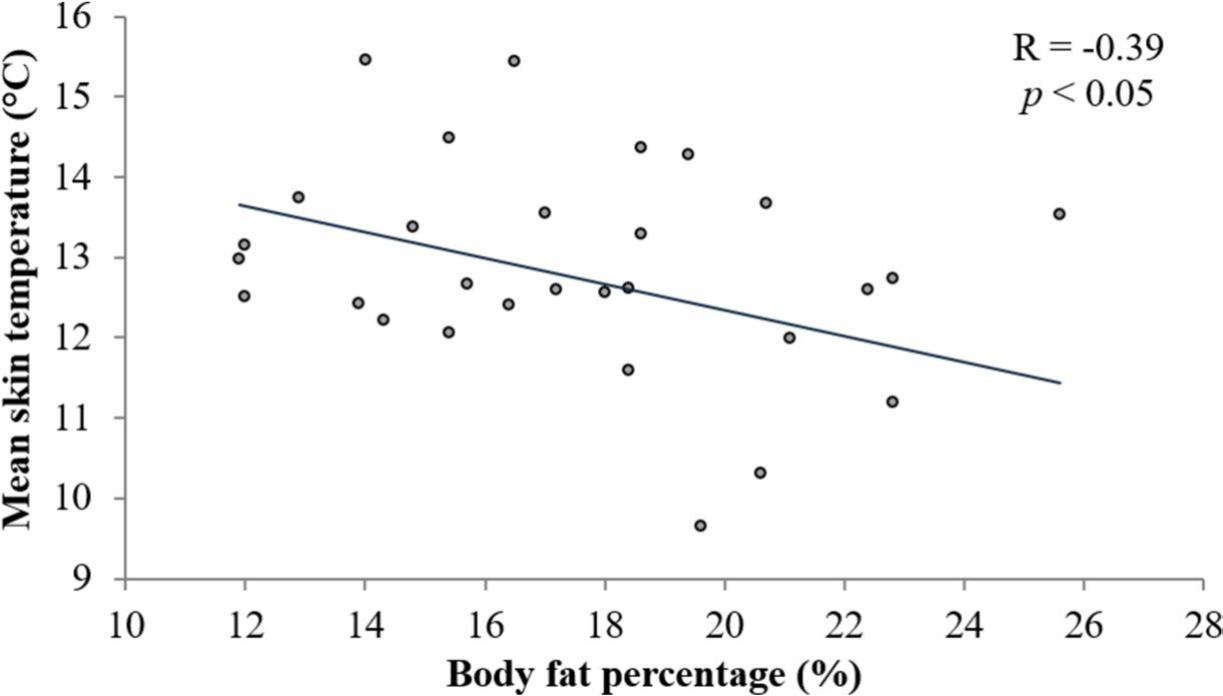
Scatter plot of body fat percentage and mean skin temperature following WBC exposure

During WBC exposure, ANOVA analysis highlighted significant variations in MsT°. For both groups, a significant MsT° decrease was observed from 30 s (*p* < 0.001) until 4 min of exposure (*p* < 0.001), as compared to pre-exposure value. Additionally, our analysis revealed a significant difference in the MsT° values between normal weight and overweight groups at 3 min 30 s (*p* < 0.05) and 4 min (*p* < 0.05) of exposure (Fig. 2). These results highlighted that young men with a normal weight reached the analgesic threshold after 4 min of exposure (*p* < 0.05), whereas overweight participants reached it earlier, at 3 min 30 s (*p* < 0.05). In addition, Pearson correlation indicated a significant and negative relationship (*R* =  − 0.39; *t* =  − 2.24; *p* < 0.05) between MsT° after 4 min of WBC exposure and body fat percentage (%) (Fig. 3).

#### Segmental analysis

**Fig. 4:**
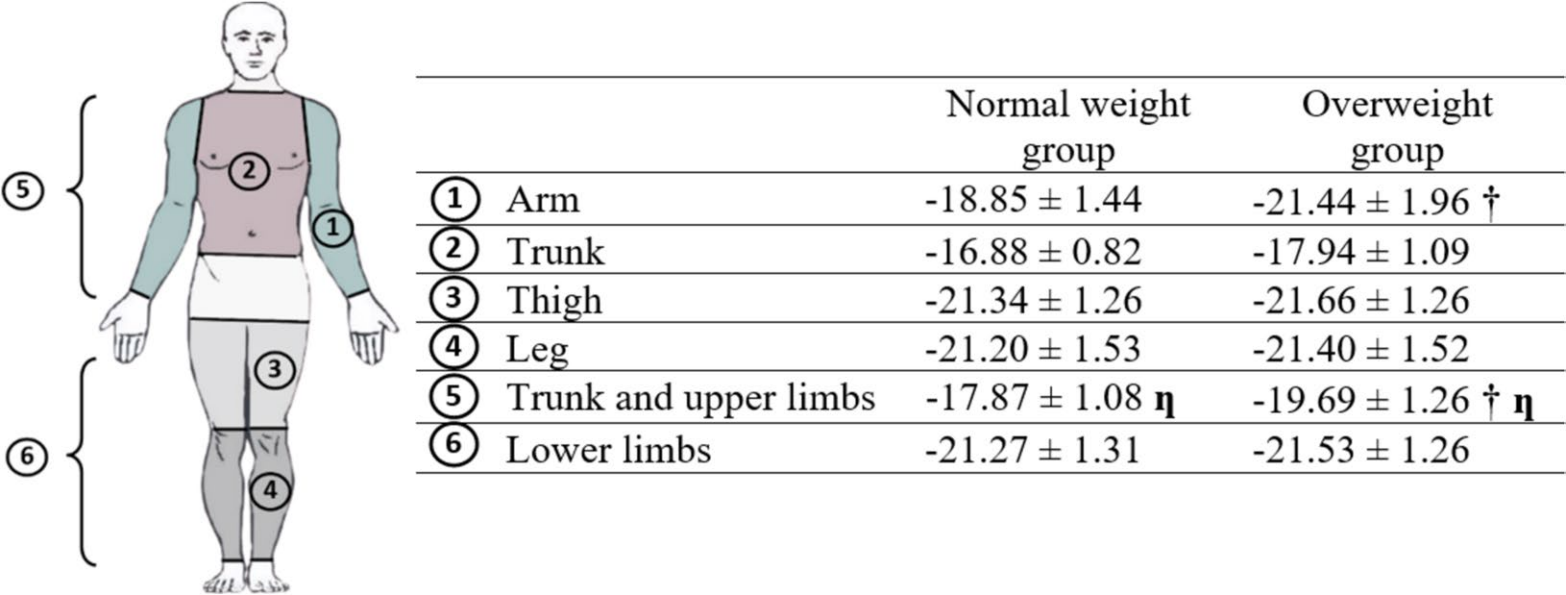
Mean value changes of skin temperature in selected body areas immediately after WBC exposure. †: Significant difference compared to normal weight group (*p* < 0.001); ƞ: significant difference between upper and lower body segments (*p* < 0.001); data are presented as mean ± SD

Immediately after 4 min of WBC exposure, overweight participants experienced a more pronounced decrease in MsT° within the upper body (*p* < 0.001), specifically in the arms (*p* < 0.001), compared to the normal weight group (Fig. 4). Segmental variation analysis demonstrated a greater reduction in MsT° in the lower limbs compared to the trunk and upper limbs (*p* < 0.001) for all groups.

### Mean skin temperature changes following exposure

**Fig. 5:**
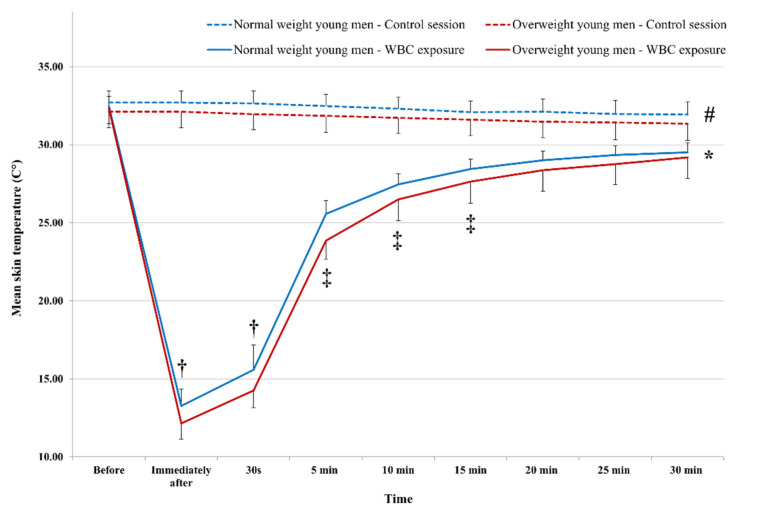
BMI effect on mean skin temperature changes following WBC exposure. *: Significant difference compared to pre-exposure value, at all measurement times, for both groups (*p* < 0.001); ‡: significant difference compared to the preceding measurement, for both groups (*p* < 0.001); #: significant difference compared to WBC exposure, at all measurement times, for both groups (*p* < 0.001); †: significant difference between groups (*p* < 0.001); data are presented as mean ± SD

Following WBC exposure, a significant increase in MsT° was observed from 30 s to 15 min post-exposure across all groups (*p* < 0.001) (Fig. 5). The recovery kinetics can be segmented into several distinct temporal phases. Initially, from 0 to 5 min, the increase in MsT° is approximately + 12.30 °C for normal weight and + 11.71 °C for overweight participants, indicating a rapid rewarming. Subsequently, between 5 and 15 min post-exposure, the MsT° increment is more moderate, around + 2.88 °C and + 3.79 °C for normal weight and overweight participants respectively. Beyond 15 to 30 min post-exposure, no further significant increase in MsT° was noted (normal weight: + 1.07 °C; overweight: + 1.54 °C). However, the measured temperatures remained considerably lower than pre-exposure values (normal weight: + 1.07 °C; overweight: + 1.54 °C; *p* < 0.001). The analysis revealed significantly lower values of MsT° following 4 min of WBC exposure compared to the control session persisting until 30 min (*p* < 0.001). Additionally, significant effects of BMI on MsT° were only observed at 30 s following 4 min WBC (*p* < 0.001).

## Discussion

The current study aimed to explore how body composition might affect cold-induced thermal responses in the context of WBC. The main objective was to determine the optimal exposure duration required to reach the analgesic threshold of 13.6 °C, a temperature suspected to trigger the beneficial effects of cold therapy [25, 26]. In fact, in response to changes in the outside environment or internal body temperature, the blood vessels in the skin can either dilate or constrict. This plays a crucial role in maintaining a steady body temperature by managing the loss or conservation of body heat through the skin surface [27]. Overall, our results demonstrated that, during the WBC sessions, different MsT° patterns were observed, which were affected by participants’ BMI. Normal weight participants reached the threshold in 4 min, whereas overweight participants reached it in 3 min 30 s. Our finding suggests that optimal WBC dosage may differ among different BMI categories. Indeed, overweight participants needed shorter exposure duration to reach the threshold temperature of 13.6 °C. To our knowledge, no prior study has specifically investigated the optimal WBC duration based on the analgesic threshold of 13.6 °C. Previous research has primarily focused on other parameters such as skin temperature magnitude, cardiovascular responses, and subjective thermal comfort. For instance, Selfe et al. examined the effects of different WBC durations on elite rugby league players with normal weight, focusing on inflammatory markers, tissue oxygenation, and skin and core temperature. They concluded that a 2-min exposure at − 135 °C following 30 s of pre-cooling at − 60 °C was optimal for inducing physiological responses without negative effects. The authors reported that a 2-min exposure is sufficient to induce beneficial physiological and perceptual changes that surpass those achieved with a 1-min exposure, while avoiding the negative effects associated with a 3-min exposure. Similarly, Fonda et al. [7] investigated thermal and cardiovascular responses in healthy young normal weight men following different exposure duration (1 min 30 s, 2 min, 2 min 30 s, and 3 min), at temperatures between − 130 and − 170 °C. They found that skin temperature decreased significantly with longer exposures up to 2 min 30 s. However, longer exposure did not significantly affect the thermal response. Due to varying evaluation criteria, recommended optimal durations differ widely. Notably, previous studies did not base their recommendations on the thermal threshold of 13.6 °C. The novelty of the present study lies in identifying the duration needed to reach this analgesic threshold, which is crucial for triggering the cold-induced responses. Additionally, the duration of exposure also varies due to different cryostimulation protocols. In our study, we used a temperature of − 50 °C with a wind speed of 2.3 m/s, unlike other studies that employed much lower temperatures. These variations in temperature settings and methodologies may explain the different recommended durations. Moreover, previous studies did not compare different BMI categories to personalize the duration according to this factor. Many studies have identified different thermal responses among BMI categories, highlighting the need for tailored WBC protocols. For example, Claessens-van Ooijen et al. [15] and Cholewka et al. [9] both demonstrated different thermal responses in individuals based on their body composition, emphasizing the importance of considering BMI in WBC treatments.

The initial and sustained response to whole-body cold exposure is marked by cutaneous vasoconstriction [1]. This reflex mechanism serves as the major autonomic reaction to reduce convective heat transmission and protect core temperature [28]. Responses to cold exposure can be influenced by body composition, specifically body fat. Adipose tissue exhibits decreased dermic thermal conductivity and increased insulating proprieties, effectively forming a barrier against conductive heat transfer [29, 30]. Consequently, individuals with higher levels of subcutaneous fat had greater protection against heat loss. In agreement, overweight individuals who have higher levels of adiposity tend to exhibit a more pronounced degree of skin cooling. In the same way, Claessens-van Ooijen et al. [15] have shown that the increase in heat production (thermogenesis) during cold exposure could be three times greater in lean individuals compared to overweight participants. These findings are further supported by the observed negative correlation between MsT° and body fat percentage. Hammond et al. [20] have previously observed a strong negative relationship between change in skin temperature and body fat percentage supporting the hypothesis that a higher body fat percentage may facilitate a more pronounced reduction in skin temperature.

Segmentary analysis revealed that, regardless of BMI categories, greater MsT° changes were observed in lower limbs compared to the trunk and upper limbs, as shown in prior cryostimulation studies [20, 31, 32]. Cold air, characterized by its high density, tends to accumulate at the bottom of the cabin, while warmer air, with its lower density, naturally rises to the upper section [33]. Consequently, in static air cryostimulation chambers, the vertical temperature gradient could lead to heterogeneity in skin temperature. However, the temperature distribution may be more homogenous in ventilated cold chamber even though a non-uniform ventilation may maintain temperature gradient in the chamber. Further investigation into the limb-specific differences in skin temperature reveals that these disparities may also be attributed to the different vascular responses to cold exposure. Notably, the cutaneous vasoconstrictor response is greater in the legs than in the arms due to higher α1- and α2-adrenoceptor reactivity [34]. In response to cold, the vasoconstriction process plays an important role in reducing skin temperature by effectively reducing blood flow to the skin surface. Furthermore, segmentary analysis showed a more pronounced decrease in skin temperature of the arms in overweight compared to normal weight individuals, which may be linked to fat distribution. Specifically, in regions such as the arms, overweight individuals tend to have a higher amount of body fat, which may provide enhanced insulation. This increased insulation effect could result in a more marked decrease in skin temperature when exposed to cold.

Our study’s findings could be contextualized within the framework of physiological anthropology, particularly Allen’s and Bergmann’s rules, associated to body shape and size adaptations in response to climate. Allen’s rule suggests that endothermic animals in colder climates tend to have shorter appendages (e.g., limbs, ears) to minimize heat loss [35]. This concept may explain the pronounced cooling effects observed in our participants’ lower limbs, where temperatures were cooler compared to other body parts. According to Allen’s rule, animals with shorter limbs have a reduced surface area, which helps them conserve body heat and survive in cold environments [36]. Our findings support this rule by demonstrating a more pronounced decrease in lower limb skin temperature, which present a higher surface area.

Bergmann’s rule states that animals in colder climates tend to have larger body sizes to reduce heat loss through a smaller surface area-to-volume ratio [37]. This principle supports our observation that individuals with higher BMIs exhibited different thermal responses compared to those with normal weight. The larger body mass in overweight participants provides better insulation [38], potentially affecting their skin temperature response to WBC exposure. In fact, overweight individuals, with a higher percentage of body fat, may exhibit slower heat dissipation. However, it is important to note that our study primarily examined the effect of body fat and BMI, rather than comparing individuals of different body sizes. Therefore, while our results align with the general principles of Bergmann’s rule, they should be interpreted with caution and considered as indicative rather than conclusive evidence of the influence of body size on thermal regulation and cold tolerance. To fully apply the Bergmann’s rule, future research should categorize participants based on body morphology (surface area) rather than solely on BMI. In summary, our results align with Allen’s and Bergmann’s rules, suggesting that body size and composition may significantly influence thermal regulation and cold tolerance. The observed differences in skin temperature responses between normal weight and overweight participants may reflect underlying physiological adaptations to temperature regulation consistent with these anthropological principles.

Following WBC exposure, the immediate increase in skin temperature likely reflects a reactive hyperemia response, a well-documented phenomenon where blood flow increases following cold exposure. WBC induces significant skin and peripheral tissue cooling, effectively reducing peripheral blood flow due to vasoconstriction [1]. After cold exposure, there is a rebound effect where blood vessels dilate (vasodilation) significantly, leading to a marked increase in blood flow to the skin and peripheral tissues [39, 40]. This peripheral vasodilation is a protective reflex to counteract the vasoconstriction during WBC and rapidly rewarm the skin, raising skin temperature. Reactive hyperemia is essentially the reflex of restoring normal blood flow and oxygen delivery to tissues that experienced reduced perfusion (vasoconstriction) [32]. After the pronounced and immediate increase in skin temperature following cold exposure, a more moderate rate of skin rewarming was observed. This moderation can be primarily attributed to temperature gradient between the skin and the environment (ambient temperature). Initially, this gradient was important due to the cold-induced lowering of skin temperature, facilitating rapid heat loss to the environment. Five minutes following exposure, as skin temperature rises, the gradient diminishes, resulting to a reduced difference in temperature between the skin and the ambient air. Consequently, the rate of heat transfer from the body to the environment slows down [41], resulting in a deceleration of the rewarming rate. Beyond 15 min post-exposure, the increase in MsT° reaches a plateau, indicating that the body has entered a phase of thermal balance [41, 42]. This equilibrium indicates that the rates of heat production and loss within the body have balanced out [41]. The plateau phase does not necessarily mean that the skin temperature has reached pre-WBC but suggests that complete recovery to reach baseline skin temperature takes longer than 30 min post-exposure. This may be due to several factors, including the extend of the initial temperature decrease and the ambient conditions in which the rewarming is taking place.

Following WBC, a rapid skin temperature increase was observed. Cryostimulation is known to significantly reduce inflammation, oxidative stress, and nerve conduction velocity which have an impact to induce analgesia. In the clinical context, particularly for patients experiencing delayed onset muscle soreness or pain, which retain them to exercise and move, it could be of interest to place cryostimulation before exercising. Therapeutic techniques such as joint mobilization and muscle stretching could be more tolerable following cryostimulation in patients with pain-related illnesses such as fibromyalgia and arthritis. Given the fast rewarming observed after cold exposure, it may be advisable [43], especially in clinical context, to initiate physical exercises and mobilization quite quickly after cold exposure. This recommendation aligns with Belitsky et al.’s suggestions regarding local cryotherapy [43]. Furthermore, our results revealed that, regardless of individual characteristics, at 30 min post-exposure, MsT° remained significantly lower than baseline values. These findings align with those of Westerlund et al. [44]. Additionally, Klimek et al. [45] reported that, following WBC, thigh surface temperature did not return to baseline levels until 90 min after exposure. These insights lead to the recommendation that practitioners should be attentive to the post-cooling period.

## Strengths, limitations, and perspectives

Our research addresses a notable gap in the literature by providing the optimal duration of WBC exposure to reach the targeted skin temperature of 13.6 °C with considering BMI. This study uncovers that individuals exhibit variations in skin cold-induced thermal responses, offering valuable insights for the development of personalized WBC protocols. Moreover, it provides guidance for healthcare professionals, trainers, practitioners, athletes, patients, and subjects practicing WBC for quality of life purposes to optimize benefits and minimize risks, ensuring the safe administration of WBC. Additionally, our findings can play crucial role in optimizing recovery strategies after strenuous exercise, potentially leading to enhanced athletic performance and reduced injury risk. In light of the presented findings, it would be insightful for future investigations to explore other factors that might influence thermal responses, such as age and sex. Such considerations would optimize the use of WBC for a wide range of the population.

It is important to note that our work is not free of limitations. Firstly, our results are specific to the type of cryo-chamber used in this research, which has a particular temperature setting (− 50 °C with a wind speed of 2.3 m/s). With other types of cryo-chambers, the timing for reaching the targeted temperature of 13.6 °C may be different and should be adjusted if needed. Nevertheless, the BMI will still need be considered as in our work. Additionally, our research design only involved normal weight and overweight participants. The inclusion of obese or underweight individuals would certainly have allowed a more complete and more relevant analysis but would have required additional ethical considerations and medical setting. This study should be completed by incorporating these BMI categories in future research to comprehensively understand the effects of BMI on WBC outcomes. Furthermore, the lack of agreed guidelines for calculating the MsT° formula, the regions selected for this study may not be directly comparable to those used in other researches. It is worth noting that our choice of formula was based on areas directly exposed to cryostimulation, deliberately excluding protected regions (face and hands…). An additional study limitation arises from cold-induced thermal perception which prevents participant blinding.

## Conclusion

This study highlights the necessity of considering body composition when assessing the effects of WBC on MsT° changes. This study underscores that individuals exhibit variations in cold-induced thermal responses, emphasizing the need for personalized approaches tailored on individual characteristics. In fact, our findings showed that men with a normal weight reached the threshold temperature of 13.6 °C in 4 min, whereas overweight people reached it in 3 min 30 s with this kind of WBC technology. Understanding the impact of BMI on cold exposure outcomes can help in optimizing WBC treatments and maximizing potential benefits.

## Data Availability

Data supporting the conclusions of this article can be accessed by contacting the corresponding author.

## Abbreviations

ANOVA: Analysis of variance
BMI: Body mass index
MsT°: Mean skin temperature
WBC: Whole-body cryostimulation

## References

[1] Bouzigon R, Grappe F, Ravier G, Dugue B. Whole- and partial-body cryostimulation/cryotherapy: current technologies and practical applications. J Therm Biol. 2016;61:67–81. 27712663 10.1016/j.jtherbio.2016.08.009

[2] Douzi W, Dupuy O, Tanneau M, Boucard G, Bouzigon R, Dugué B. 3-min whole body cryotherapy/cryostimulation after training in the evening improves sleep quality in physically active men. Eur J Sport Sci. 2019;19:860–7. 30551730 10.1080/17461391.2018.1551937

[3] Dupuy O, Douzi W, Theurot D, Bosquet L, Dugué B. An evidence-based approach for choosing post-exercise recovery techniques to reduce markers of muscle damage, soreness, fatigue, and inflammation: a systematic review with meta-analysis. Front Physiol. 2018;9:1–15. 29755363 10.3389/fphys.2018.00403 PMC5932411

[4] Ntoumani M, Dugué B, Rivas E, Gongaki K. Thermoregulation and thermal sensation during whole-body water immersion at different water temperatures in healthy individuals: a scoping review. J Therm Biol. 2023;112: 103430. 36796887 10.1016/j.jtherbio.2022.103430

[5] Louis J, Theurot D, Filliard J-R, Volondat M, Dugué B, Dupuy O. The use of whole-body cryotherapy: time- and dose-response investigation on circulating blood catecholamines and heart rate variability. Eur J Appl Physiol. 2020;120:1733–43. 32474683 10.1007/s00421-020-04406-5 PMC7340648

[6] Bleakley C, Bieuzen F, Davison G, Costello J. Whole-body cryotherapy: empirical evidence and theoretical perspectives. Open Access J Sports Med. 2014;5:25–36. 24648779 10.2147/OAJSM.S41655 PMC3956737

[7] Fonda B, De Nardi M, Sarabon N. Effects of whole-body cryotherapy duration on thermal and cardio-vascular response. J Therm Biol. 2014;42:52–5. 24802149 10.1016/j.jtherbio.2014.04.001

[8] Selfe J, Alexander J, Costello JT, May K, Garratt N, Atkins S, et al. The effect of three different (-135°C) whole body cryotherapy exposure durations on elite rugby league players. He X, editor. PLoS ONE. 2014;9:e86420. 10.1371/journal.pone.0086420 PMC3906033 24489726

[9] Cholewka A, Stanek A, Sieroń A, Drzazga Z. Thermography study of skin response due to whole-body cryotherapy: thermography study of skin response due to whole-body cryotherapy. Skin Res Technol. 2012;18:180–7. 21507075 10.1111/j.1600-0846.2011.00550.x

[10] Costello JT, McInerney CD, Bleakley CM, Selfe J, Donnelly AE. The use of thermal imaging in assessing skin temperature following cryotherapy: a review. J Therm Biol. 2012;37:103–10.

[11] Legrand FD, Dugué B, Costello J, Bleakley C, Miller E, Broatch JR, et al. Evaluating safety risks of whole-body cryotherapy/cryostimulation (WBC): a scoping review from an international consortium. Eur J Med Res. 2023;28:387. 37770960 10.1186/s40001-023-01385-z PMC10537204

[12] Chesterton LS, Foster NE, Ross L. Skin temperature response to cryotherapy. Arch Phys Med Rehabil. 2002;83:543–9. 11932859 10.1053/apmr.2002.30926

[13] Bleakley CM, Hopkins JT. Is it possible to achieve optimal levels of tissue cooling in cryotherapy? Physical Therapy Reviews. 2010;15:344–50.

[14] Algafly AA, George KP. The effect of cryotherapy on nerve conduction velocity, pain threshold and pain tolerance. Br J Sports Med. 2007;41:365–9. 17224445 10.1136/bjsm.2006.031237 PMC2465313

[15] Claessens-van Ooijen AMJ, Westerterp KR, Wouters L, Schoffelen PFM, van Steenhoven AA, van Marken Lichtenbelt WD. Heat production and body temperature during cooling and rewarming in overweight and lean men. Obesity. 2006;14:1914–20. 17135606 10.1038/oby.2006.223

[16] Castellani JW, Young AJ, Sawka MN, Pandolf KB. Human thermoregulatory responses during serial cold-water immersions. J Appl Physiol. 1985;1998(85):204–9. 10.1152/jappl.1998.85.1.204 9655776

[17] Polidori G, Elfahem R, Abbes B, Bogard F, Legrand F, Bouchet B, et al. Preliminary study on the effect of sex on skin cooling response during whole body cryostimulation (−110 °C): modeling and prediction of exposure durations. Cryobiology. 2020;97:12–9. 33130106 10.1016/j.cryobiol.2020.10.014

[18] Otte JW, Merrick MA, Ingersoll CD, Cordova ML. Subcutaneous adipose tissue thickness alters cooling time during cryotherapy. Arch Phys Med Rehabil. 2002;83:1501–5. 12422316 10.1053/apmr.2002.34833

[19] Valensi P, Smagghue O, Pariès J, Velayoudon P, Lormeau B, Attali JR. Impairment of skin vasoconstrictive response to sympathetic activation in obese patients: influence of rheological disorders. Metabolism. 2000;49:600–6. 10831169 10.1016/s0026-0495(00)80034-7

[20] Hammond LE, Cuttell S, Nunley P, Meyler J. Anthropometric characteristics and sex influence magnitude of skin cooling following exposure to whole body cryotherapy. Biomed Res Int. 2014;2014: 628724. 25061612 10.1155/2014/628724 PMC4100349

[21] Weir CB, Jan A. BMI classification percentile and cut off points. StatPearls. Treasure Island (FL): StatPearls Publishing; 2024. Available from: http://www.ncbi.nlm.nih.gov/books/NBK541070/ Cited 2024 Feb 1. 31082114

[22] Mohajan D, Mohajan H. A study on body fat percentage for physical fitness and prevention of obesity: a two compartment model. 2023;2:1–10.

[23] Bouzigon R, Arfaoui A, Grappe F, Ravier G, Jarlot B, Dugue B. Validation of a new whole-body cryotherapy chamber based on forced convection. J Therm Biol. 2017;65:138–44. 28343567 10.1016/j.jtherbio.2017.02.019

[24] Ramanathan NL. A new weighting system for mean surface temperature of the human body. J Appl Physiol. 1964;19:531–3. 14173555 10.1152/jappl.1964.19.3.531

[25] Bugaj R. The cooling, analgesic, and rewarming effects of ice massage on localized skin. Phys Ther. 1975;55:11–9. 1088989 10.1093/ptj/55.1.11

[26] Leppäluoto J, Westerlund T, Huttunen P, Oksa J, Smolander J, Dugué B, et al. Effects of long-term whole-body cold exposures on plasma concentrations of ACTH, beta-endorphin, cortisol, catecholamines and cytokines in healthy females. Scand J Clin Lab Invest. 2008;68:145–53. 18382932 10.1080/00365510701516350

[27] Sawasaki N, Iwase S, Mano T. Effect of skin sympathetic response to local or systemic cold exposure on thermoregulatory functions in humans. Auton Neurosci. 2001;87:274–81. 11476289 10.1016/S1566-0702(00)00253-8

[28] Johnson JM, Minson CT, Kellogg DL. Cutaneous vasodilator and vasoconstrictor mechanisms in temperature regulation. Compr Physiol. 2014;4:33–89. 24692134 10.1002/cphy.c130015

[29] Castellani JW, Young AJ. Human physiological responses to cold exposure: acute responses and acclimatization to prolonged exposure. Auton Neurosci. 2016;196:63–74. 26924539 10.1016/j.autneu.2016.02.009

[30] Savastano DM, Gorbach AM, Eden HS, Brady SM, Reynolds JC, Yanovski JA. Adiposity and human regional body temperature. Am J Clin Nutr. 2009;90:1124–31. 19740972 10.3945/ajcn.2009.27567 PMC2762153

[31] Savic M, Fonda B, Sarabon N. Actual temperature during and thermal response after whole-body cryotherapy in cryo-cabin. J Therm Biol. 2013;38:186–91.

[32] Hohenauer E, Costello JT, Stoop R, Küng UM, Clarys P, Deliens T, et al. Cold-water or partial-body cryotherapy? Comparison of physiological responses and recovery following muscle damage. Scand J Med Sci Sports. 2018;28:1252–62. 29130570 10.1111/sms.13014

[33] Polidori G, Taiar R, Legrand F, Beaumont F, Murer S, Bogard F, et al. Infrared thermography for assessing skin temperature differences between partial body cryotherapy and whole body cryotherapy devices at −140 °C. Infrared Phys Technol. 2018;93:158–61.

[34] Yamazaki F, Yuge N. Limb-specific differences in the skin vascular responsiveness to adrenergic agonists. J Appl Physiol. 1985;2011(111):170–6. 10.1152/japplphysiol.00068.2011 21527669

[35] Allen JA. The influence of physical conditions in the genesis of species. Radic Rev. 1877;1:108–40.

[36] McQueen A, Klaassen M, Tattersall GJ, Atkinson R, Jessop R, Hassell CJ, et al. Thermal adaptation best explains Bergmann’s and Allen’s rules across ecologically diverse shorebirds. Nat Commun. 2022;13:4727. 35953489 10.1038/s41467-022-32108-3 PMC9372053

[37] Blackburn T, Gaston K, Loder N. Geographic gradients in body size: a clarification of Bergmann’s rule. Biodiversity research Diversity Distrib. 1999;5:165–74.

[38] Steegmann AT, Cerny FJ, Holliday TW. Neandertal cold adaptation: physiological and energetic factors. Am J Hum Biol. 2002;14:566–83. 12203812 10.1002/ajhb.10070

[39] Gregson W, Black MA, Jones H, Milson J, Morton J, Dawson B, et al. Influence of cold water immersion on limb and cutaneous blood flow at rest. Am J Sports Med. 2011;39:1316–23. 21335348 10.1177/0363546510395497

[40] Costello J, McNamara PM, O’Connell ML, Algar LA, Leahy MJ, Donnelly AE. Tissue viability imaging of skin microcirculation following exposure to whole body cryotherapy (-110°C) and cold water immersion (8°C). Archives of Exercise in Health and Disease. 2014;4:243–50.

[41] Arnold M, Millar R. Children’s and lay adults’ views about thermal equilibrium. Int J Sci Educ. 1994;16:405–19.

[42] Taylor NAS, Tipton MJ, Kenny GP. Considerations for the measurement of core, skin and mean body temperatures. J Therm Biol. 2014;46:72–101. 25455943 10.1016/j.jtherbio.2014.10.006

[43] Belitsky RB, Odam SJ, Hubley-Kozey C. Evaluation of the effectiveness of wet ice, dry ice, and cryogen packs in reducing skin temperature. Phys Ther. 1987;67:1080–4. 3602101 10.1093/ptj/67.7.1080

[44] Westerlund T, Oksa J, Smolander J, Mikkelsson M. Thermal responses during and after whole-body cryotherapy (−110°C). J Therm Biol. 2003;28:601–8.

[45] Klimek A, Lubkowska A, Szyguła Z, Frączek B, Chudecka M. The influence of single whole body cryostimulation treatment on the dynamics and the level of maximal anaerobic power. Int J Occup Med Environ Health. 2011;24:184–91. 21590430 10.2478/s13382-011-0017-z

